# Screening large-scale association study data: exploiting interactions using random forests

**DOI:** 10.1186/1471-2156-5-32

**Published:** 2004-12-10

**Authors:** Kathryn L Lunetta, L Brooke Hayward, Jonathan Segal, Paul Van Eerdewegh

**Affiliations:** 1 Oscient Pharmaceuticals, Inc. (formerly Genome Therapeutics Corporation), Waltham, Massachusetts, USA; 2 Department of Biostatistics, Boston University School of Public Health, Boston, Massachusetts, USA; 3 Genizon BioSciences Inc., Montreal, Quebec, Canada; 4 Department of Psychiatry, Harvard Medical School, Boston, Massachusetts, USA

## Abstract

**Background:**

Genome-wide association studies for complex diseases will produce genotypes on hundreds of thousands of single nucleotide polymorphisms (SNPs). A logical first approach to dealing with massive numbers of SNPs is to use some test to screen the SNPs, retaining only those that meet some criterion for futher study. For example, SNPs can be ranked by p-value, and those with the lowest p-values retained. When SNPs have large interaction effects but small marginal effects in a population, they are unlikely to be retained when univariate tests are used for screening. However, model-based screens that pre-specify interactions are impractical for data sets with thousands of SNPs. Random forest analysis is an alternative method that produces a single measure of importance for each predictor variable that takes into account interactions among variables without requiring model specification. Interactions increase the importance for the individual interacting variables, making them more likely to be given high importance relative to other variables. We test the performance of random forests as a screening procedure to identify small numbers of risk-associated SNPs from among large numbers of unassociated SNPs using complex disease models with up to 32 loci, incorporating both genetic heterogeneity and multi-locus interaction.

**Results:**

Keeping other factors constant, if risk SNPs interact, the random forest importance measure significantly outperforms the Fisher Exact test as a screening tool. As the number of interacting SNPs increases, the improvement in performance of random forest analysis relative to Fisher Exact test for screening also increases. Random forests perform similarly to the univariate Fisher Exact test as a screening tool when SNPs in the analysis do not interact.

**Conclusions:**

In the context of large-scale genetic association studies where unknown interactions exist among true risk-associated SNPs or SNPs and environmental covariates, screening SNPs using random forest analyses can significantly reduce the number of SNPs that need to be retained for further study compared to standard univariate screening methods.

## Background

Genome-wide association studies for complex diseases such as asthma, schizophrenia, diabetes, and hypertension will soon produce genotypes on hundreds of thousands of single nucleotide polymorphisms (SNPs). Due to the large number of SNPs tested and the potential for both genetic and environmental interactions, determining which SNPs modify the risk of disease is a methodological challenge. While the number of genotypes produced by candidate gene approaches will be somewhat less daunting, on the order of hundreds to thousands of SNPs, it will still be a considerable challenge to weed out the noise and identify the SNPs contributing to complex traits.

A logical first approach to dealing with massive numbers of SNPs is to first conduct univariate association tests on each individual SNP, in order to screen-out those with no evidence for disease association. The primary goal of such a procedure is not to prove that a particular variant or set of variants influences disease risk, but to prioritize SNPs for further study. Using a univariate test at this stage will result in low power for SNPs with very small marginal effects in the population, even if the SNPs have large interaction effects. Of course, in addition to taking all individual SNPs, all SNP pairs could also be tested for association. However, when dealing with multiple thousands of SNPs at the outset, such an approach is cumbersome, and raises the question of where to stop: why not all sets of three, four, or even five SNPs as well?

Many model-building methods exist for dealing with large numbers of predictors. For example, stochastic search variable selection (SSVS) [1], a form of Bayesian model selection, has been explored as a tool to discover joint effects of multiple loci in the context of genetic linkage studies [2-4]. However, these methods are limited in the number of predictors that can be included at one time, causing some researchers to resort to a two-stage approach, in which only main effects are considered in a first stage, and interactions between loci with strong main effects are considered in a second stage. This approach can lead to the loss of important interactions with only weak main effects.

Multivariate adaptive regression splines (MARS) models have also been explored in the context of genetic linkage and association studies [5,6] with some degree of success. However, these and other model selection methods appear to be limited in the number of predictors that can reasonably be accommodated in one analysis, and the types of possible interactions that are allowed must be specified in advance. They are not suited to the initial task of identifying from a massive set of SNPs a subset for further analyses.

Combinatorial partitioning and multifactor dimensionality reduction [7-10] are closely related methods developed specifically to detect higher-order interactions among polymorphisms that predict trait variation. However, these methods are meant to identify interactions among small sets of SNPs, and have minimal power in the presence of genetic heterogeneity [10]. They are therefore inappropriate for use as a screening tool for searching through thousands of SNPs to identify those contributing to phenotypes in the context of whole-genome association studies. The problem remains: how do we reasonably weed down from thousands or hundreds of thousands of SNPs to a number that can be used by available modeling methods, without losing the interactions that we hope to model in the first place?

An additional concern to be considered is genetic heterogeneity. We define genetic heterogeneity to mean that there are multiple possible ways to acquire a disease or trait that can involve different subsets of genes. Traditional regression models are limited in their ability to deal with underlying genetic heterogeneity (see, *e.g*., [11]). If genetic heterogeneity also leads to phenotypic heterogeneity, then methods that classify individuals into phenotypic subgroups for further analysis can be successful. Likewise, if heterogeneity in genetic etiology is primarily due to ethnic background, sub-dividing samples by self-reported ethnicity or genetically defined subgroups can be a powerful antecedent to data analyses for the identification of complex disease genes. However, even in the realm of Mendelian genetic diseases, heterogeneity is rarely so simple. For example, multiple polymorphisms in each of two different genes are responsible for familial breast cancer in the relatively homogeneous sub-population of Ashkenazi Jewish women [12]. When the root of the heterogeneity is not known *a priori*, traditional regression models, which lump all individuals into a single group and estimate average effects over the entire sample, are unlikely to successfully identify the genetic causes of diseases.

### Classification trees and random forests

Tree-based methods consist of non-parametric statistical approaches for conducting regression and classification analyses by recursive partitioning (see, e.g., Hastie et al. [13]). These methods can be very efficient at selecting from large numbers of predictor variables such as genetic polymorphisms those that best explain a phenotype. Tree methods are useful when predictors may be associated in some non-linear fashion, as no implicit assumptions about the form of underlying relationships between the predictor variables and the response are made. They are well-adapted to dealing with some types of genetic heterogeneity, as separate models are automatically fit to subsets of data defined by early splits in the tree.

The ease of interpretation of classification trees, along with their flexibility in accommodating large numbers of predictors and ability to handle heterogeneity, has resulted in increasing interest in their application to genetic association and linkage studies. Classification trees have been adapted for use with sibling pairs to subdivide pairs into more homogenous subgroups defined by non-genetic covariates [14], thus increasing the power to detect linkage under heterogeneity [15]. They have also shown promise for the dissection of complex traits for both linkage and association [16,17], and for exploring interactions [6]. A related adaptive regression method has also shown promise in selecting a small number of predictive SNPs from a set of hundreds of potential predictors [18]. Tree methods have also been used to identify homogeneous groups of cases for further analyses [19], and as an adjunct to more traditional association methods [20].

Classification trees are grown by recursively partitioning the observations into subgroups with a more homogeneous categorical response [21]. At each node, the explanatory variable (e.g., SNP) giving the most homogeneous sub-groups is selected. Choosing alternative predictors that produce slightly sub-optimal splits can result in very different trees that have similar prediction accuracy. The Random Forests methodology [22] builds on several other methods using multiple trees to increase prediction accuracy [23-25]. A random forest is a collection of classification or regression trees with two features that distinguish it from trees built in a deterministic manner. First, the trees are grown on bootstrap samples of the observations. Second, a random selection of the potential predictors is used to determine the best split at each node. For each tree, a bootstrap sample is obtained by drawing a sample with replacement from the original sample of observations. The bootstrap sample has the same number of individuals as the original sample, but some individuals are represented multiple times, while others are left out. The left-out individuals, sometimes called "out-of-bag", are used to estimate prediction error. Because a different bootstrap sample is used to grow each tree, there is a different set of out-of-bag individuals for each tree. With a forest of classification trees, each tree predicts the class of an individual. For each individual, the predictions, or "votes", are counted across all trees for which the individual was out-of-bag, and the class with the most votes is the individual's predicted class. Random forests produce an importance score for each variable that measures its importance. This score can be used to prioritize the variables, much as p-values from test statistics are used.

Using ensembles of trees built in this manner increases the probability that some trees will capture interactions among variables with no strong main effect. Unlike variable selection methods, interactions among predictors do not need to be explicitly specified in order to be utilized by a forest of trees. Instead, any interactions between variables serve to increase the importance of the individual interacting variables, making them more likely to be given high importance relative to other variables. Thus, random forests appear to be particularly well-suited to address a primary problem posed by large scale association studies. In preliminary studies, we have shown the potential of random forests in the context of linkage analysis [26]. Other investigators are beginning to recognize the potential of the Random Forest methodology for studying SNP association [27] and classification [28].

To fully understand the basis of complex disease, it is important to identify the critical genetic factors involved, and to understand the complex relationships between genotypes, environment, and phenotypes. The few successes to date in identifying genes for complex disease suggest that despite carefully collected large samples, novel approaches are needed in the pursuit to dissect the multiple and varying factors that lead to complex human traits. Ultimately, the challenge in identifying polymorphisms that modulate the risk of complex disease is to find methods that can seamlessly handle large numbers of predictors, capitalize on and identify interactions, and tease apart the multiple heterogeneous etiologies. Here, we explore the use of the Random Forest methodology [22,29] as a screening tool for identifying SNPs associated with disease in the presence of interaction, heterogeneity, and large amounts of noise due to unassociated polymorphisms.

## Results

### Genetic models

We simulated complex diseases with sibling recurrence risk ratio for the disease (*λ*_s_) fixed at 2.0 and population disease prevalence *K*_*p *_equal to 0.10. These values are consistent with or lower than estimates from known complex genetic traits, such as Alzheimer disease, where estimates of cumulative prevalence in siblings of affected range from 30–40%, compared to a population prevalence of 10% at age 80 [30]. Such traits are understood to be caused by multiple interacting genetic and environmental factors. Our genetic models incorporate both genetic heterogeneity and multiplicative interaction as defined by Risch [31]: we simulate sets of 4, 8, 16, and 32 risk SNPs ("rSNPs") in linkage equilibrium, interacting in independent pairs or quartets to increase disease risk, and contributing equally to the overall sibling recurrence risk ratio of 2 and population disease prevalence of 0.10. For simplicity, we simulated the models such that each rSNP pair or quartet accounts for the same proportion of the genetic risk, and each SNP within a pair/quartet is responsible for an equal proportion of the genetic risk. Thus, all of the rSNPs simulated for a model have the same allele frequency and the same observed marginal effect in the population. We denote the models using the shorthand HhMm, where H = h (=2, 4, 8, 16) is the number of heterogeneous systems, and M = m (=2 or 4) is the number of multiplicatively interacting SNPs within each system. For example, 16 loci are responsible for the total *λ*_s _= 2 and *K*_*p *_= .10 for models H4M4 and H8M2, and 32 loci are responsible for models H8M4 and H16M2. Table 1 presents relevant features of our models. The Methods section describes the genetic models in more detail.

**Table 1:** Genetic models used for simulating case-control data.

	Risk SNPs							Case Genotype Correlation	
		Allele	Marginal GRR		Penetrance Factors			Within System	Between System
Model	Number	Frequency	Het	Hom	0	1	2		
H2M2	4	0.207	2.85	4.71	2.4E-02	0.51	1	0.30	-0.32
H4M2	8	0.160	1.99	2.96	3.9E-04	0.50	1	0.35	-0.12
H8M2	16	0.104	1.66	2.18	8.0E-06	0.56	1	0.32	-0.05
H16M2	32	0.069	1.46	1.78	1.4E-05	0.59	1	0.28	-0.02
H4M4	16	0.282	1.63	1.79	1.2E-08	0.79	1	0.17	-0.06
H8M4	32	0.214	1.34	1.40	2.8E-03	0.86	1	0.14	-0.02

### Simulation and analysis

All analyses were performed on 100 replicate data sets of 500 cases and 500 controls. In addition to the rSNPs contributing to the trait, we simulated noise SNPs ("nSNPs"), independent of disease status, with allele frequencies distributed equally across the range .01–.99. To simulate the results of an association study, in which we do not expect to be lucky enough to genotype all polymorphisms related to a trait, we included only a subset of the total number of rSNPs in each analysis. We denote the analysis design using the shorthand KkSsNn, where K = k is the total number of rSNPs genotyped in the analysis, S = s is the number of SNPs within each interaction system genotyped, and N = n is the total number of SNPs genotyped in the design. Thus, N-K is the total number of nSNPs in the analysis. For example, suppose the genetic model is H8M4, and the design is K4S2N100. Then out of the total of 8 × 4 = 32 rSNPs that contribute to the trait, four are genotyped: two interacting SNPs from within each of two heterogeneity systems. Six heterogeneity systems are not represented at all in the analysis. In addition to the four genotyped rSNPs, 100-4 = 96 total nSNPs are also genotyped in the design.

#### Comparison of raw and standardized importance scores

Random forests version 5 software [29] produces both raw (*I*_*T*_) and standardized (*Z*_*T*_) variable importance scores (see Methods section for definitions of the scores). Little is known about the properties of importance indices under different distributions of the predictor variables. We use simulation to investigate their properties in the context of discrete predictors such as genetic polymorphisms conferring susceptibility to a complex trait.

We first compared the raw and standardized scores, in order to determine whether one might outperform the other in screening. We considered a K4S2N100 analysis design for each genetic model described in Table 1. *I*_*T *_and *Z*_T _are highly correlated; the average correlation coefficient over 100 replicate data sets ranged from .93 (H8M4) to >.99 (H2M2 and H4M2) (Table 2). The average correlation between the ranks based on *I*_*T *_and *Z*_*T *_for the 100 SNPs over the 100 replicate data sets was 0.98 for each of the six models (Table 2). Comparing the ranks of the four rSNPs among all SNPs, neither importance measure outperforms the other for all models (Table 3). The mean ranks of the rSNPs for the two measures are significantly different only for the H16M2 and H8M4 models. For H16M2, the average rank of the rSNPs is higher for *Z*_*T *_than for *I*_*T*_. The opposite is true for H8M4 (see Table 3).

**Table 2:** Summary of the correlation between *I*_*T *_and *Z*_*T *_("raw") and rank(*I*_*T*_) and rank(*Z*_*T*_) ("rank") for four rSNPs and 96 nSNPs over 100 replicate data sets: K4S2N100 analysis design.

	H2M2		H4M2		H8M2		H4M4		H16M2		H8M4	
*r*^2^	raw	rank	raw	rank	raw	rank	raw	rank	raw	rank	raw	rank
Mean	0.996	0.983	0.990	0.982	0.975	0.982	0.957	0.982	0.964	0.982	0.933	0.982
SD	0.001	0.006	0.003	0.006	0.009	0.006	0.013	0.007	0.013	0.006	0.018	0.007
Min	0.990	0.963	0.980	0.957	0.941	0.960	0.921	0.955	0.926	0.962	0.891	0.953
Max	0.998	0.993	0.996	0.992	0.989	0.993	0.984	0.992	0.983	0.994	0.970	0.992

**Table 3:** Comparison of ranks based on *Z*_*T *_and *I*_*T *_for the four rSNPs over 100 replicate data sets: K4S2N100 analysis design.

	H2M2		H4M2		H8M2		H4M4		H16M2		H8M4	
Rank:	*Z*_*T*_	*I*_*T*_	*Z*_*T*_	*I*_*T*_	*Z*_*T*_	*I*_*T*_	*Z*_*T*_	*I*_*T*_	*Z*_*T*_	*I*_*T*_	*Z*_*T*_	*I*_*T*_
Mean	2.5	2.5	2.5	2.5	2.51	2.52	2.64	2.61	5.16	5.94	9.35	8.69
SD	1.12	1.12	1.12	1.12	1.13	1.16	1.74	1.62	8.06	8.33	13.67	13.9
Max	4	4	4	4	6	8	23	21.5	77	62.5	83	88.5
p-value*	0.94		1.00		0.77		0.12		1.32E-20		2.91E-12	

*p-value for the Wilcoxon signed-rank test comparing the rSNP ranks based on *I*_*T *_and *Z*_*T*_

#### Ranking SNPs based on Z_T _and Fisher p-value

**Figure 1:**
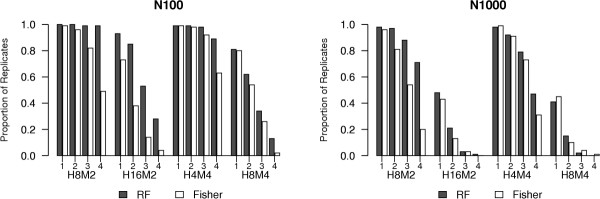
Proportion of replicates for which the most significant 1, 2, 3, and 4 SNPs are all rSNPs for K4S2N100 and K4S2N1000 analysis designs. Genetic models are listed on the plots. "RF" and "Fisher" refer to the random forest importance index Z_T _and the Fisher Exact test p-value. See text for notation description.

**Figure 2:**
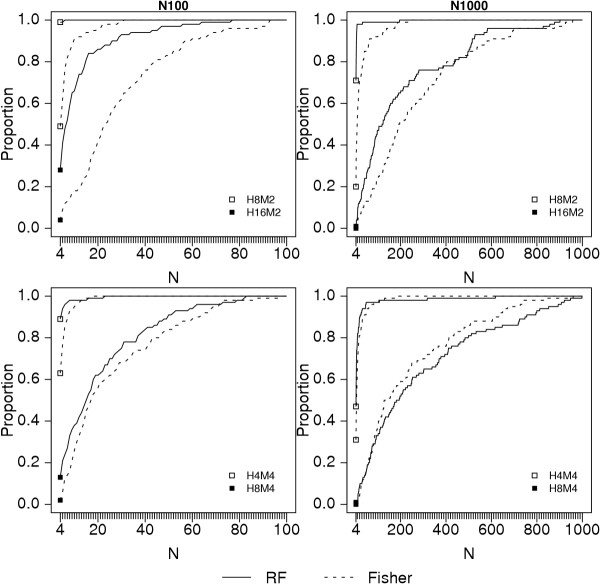
Proportion of replicates for which all rSNPs are among the top-ranking N SNPs for K4S2N100 and K4S2N1000 analysis designs. Other notation as in Figure 1.

We next compared the ranking of rSNPs by importance score (Z_T_) to ranking by Fisher Exact test p-value using K4S2N100 and K4S2N1000 analysis designs, where two SNPs from each of the first two interaction systems are in the analysis. Figure 1 shows the proportion of replicates for which the top ranked 1, top 2, top 3, and top 4 SNPs are the four genotyped rSNPs in the data set for each of the four most complex genetic models. For N100, the random forest Z_T _criterion ranks the four rSNPs as the most significant SNPs more often than the univariate Fisher Exact test association p-value under all genetic models. The difference between the random forest and association p-value ranking is less extreme for N1000. For the H8M4 genetic model, the results do not suggest that one ranking system is better than the other overall. Figure 2 shows the proportion of replicates for which all rSNPs are among the top N SNPs. In other words, it is the proportion of data sets for which none of the genotyped rSNPs are screened out, if the top ranking N SNPs are chosen for further study. For N100, a consistently higher proportion of replicates ranked using Z_T _contain all of the rSNPs. Thus, for a given probability of retaining all of the rSNPs, more SNPs can be eliminated using the Z_T _criterion than the Fisher exact test p-value. For example, for model H16M2, only 15 SNPs must be retained to have 80% probability that the 4 rSNPs are in the retained set, while 44 SNPs must be retained if the p-value criterion is used. The difference is less dramatic for H8M4: 37 SNPs give 80% probability that the four genotyped rSNPs are in the retained set for the Z_T _criterion, compared to 43 for the p-value criterion. For N1000, the advantage of the Z_T _criterion is clear for the H8M2 and H16M2 models. For H4M4, the advantage of Z_T _is minor, while for H8M4, ranking by Z_T _appears to give poorer results than the p-value criterion for the higher cutoff values of N. A second interpretation of Figure 2 is that, for any number of retained (not screened-out) SNPs, the probability that all of the genotyped rSNPs are retained is higher for the Z_T _criterion than for the univariate p-value criterion for all but the H8M4 model with 1000 total SNPs.

**Figure 3:**
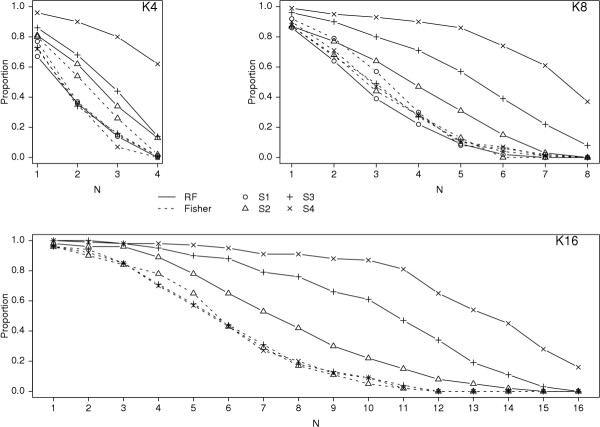
Proportion of replicates for which the most significant N SNPs are all rSNPs. H8M4 genetic model. Analysis designs include 96 noise SNPs; K and S are listed on the plots. Other notation as in Figure 1.

**Figure 4:**
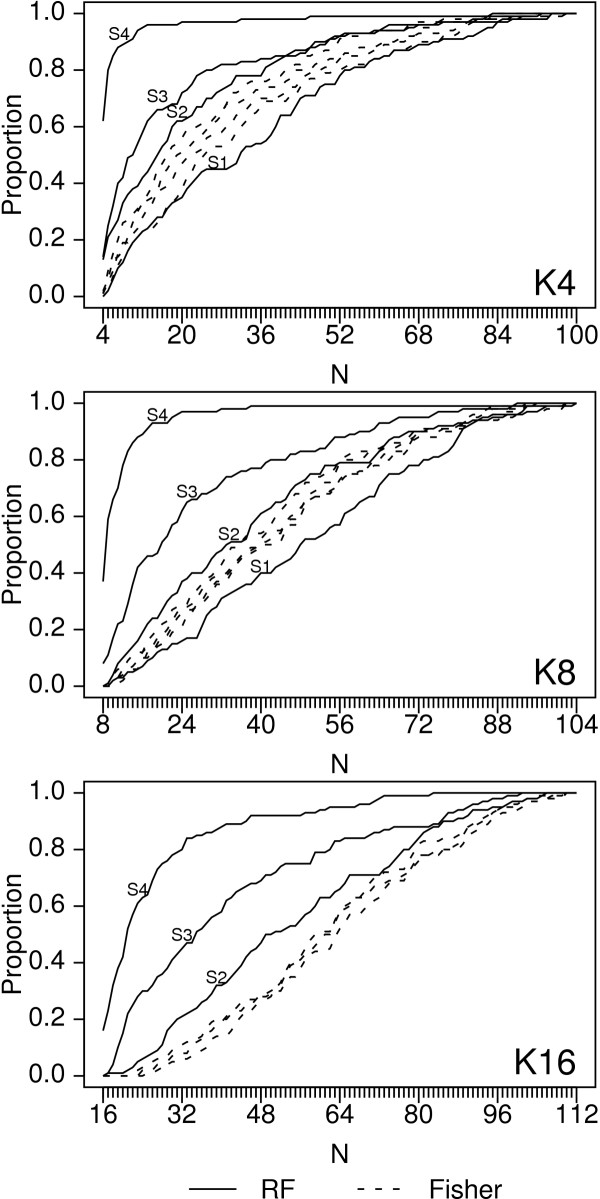
Proportion of replicates for which all rSNPs are among the top-ranking N SNPs for H8M4 genetic model. Analysis designs include 96 noise SNPs; K and S are listed on the plots.

Noticing that the analyses with all SNPs from an interacting system (e.g., the H16M2K4S2 simulations) had a more substantial improvement in ranking using Z_T _over p-value than the analyses with subsets of SNPs from an interacting system, we hypothesized that the interactions among the pairs of analyzed rSNPs influenced the improved ranking performance of the random forests over the univariate tests. To confirm this, we used the H8M4 genetic model and analyzed the data in the following manner. For a constant number of analyzed rSNPs included in the model (K = 4, 8, or 16) and a constant 96 nSNPs, we looked at the effect of increasing S, the number of rSNPs from each interaction system that were genotyped. Thus, for K8S1, along with 96 nSNPs, one SNP from each of the first 8 systems was included in the analysis, while for K8S4, all four SNPs in the first two systems were included in the analysis. For K8S3, three SNPs from the first two systems, and one from the third were included. Assuming that the random forest analysis was taking advantage of the interactions among the rSNPs, and that this was responsible for the improved performance of the random forests over the univariate tests, we expected the Fisher p-values and random forest importance Z_T _to perform similarly when only a single rSNP was genotyped from each system, and the random forests to perform increasingly better than the univariate Fisher tests as S increased from 1 to 4. Figures 3 and 4 show the results, which are consistent with this hypothesis. For the Fisher p-values, the proportion of replicates for which the N most significant SNPs were rSNPs is similar for each S. For the random forest importance Z_T_, the S = 1 analyses for each K were similar to the Fisher results, while for each increase in S, the proportion of replicates for which the N most significant SNPs were rSNPs increases (Figure 3) and the proportion of replicates for which all rSNPs are present at any cutoff point increases (Figure 4). The differences can be substantial: for the H8M4 model, with K = 4 rSNPs in the analysis, the number of most significant SNPs required to have 80% probability that all four rSNPs are included is 50, 34, 22, and 5, respectively for S1, S2, S2, and S4. We conclude that for a given number of rSNPs within a set of potential predictors, the more interacting SNPs there are, and the larger the groups of SNPs that interact, the greater the performance increase of the random forest analysis as compared to a univariate analysis.

#### Magnitude of difference

Beyond simply ranking SNPs, we may wish to use the magnitude of the difference in importance or p-value to determine which subset of top-ranked SNPs should be prioritized for further study. Thus, particularly for the cases where the rSNPs are among the top-ranked SNPs, we want to determine not just that Z_T _ranks interacting rSNPs higher than the univariate test, but also that the differences in rank correspond to differences in magnitude of Z_T _that are meaningful. In other words, we want to know how much "better" in terms of Z_T _(or p-value) the rSNPs are than the nSNPs. Toward this goal, we computed the difference between the importance Z_T _of the top ranked rSNP and the top ranked nSNP:

D_max_(Z_T_) = max_*rSNP*_(*Z*_*T*_) - max_*nSNP*_(*Z*_*T*_),

as well as the lowest ranked rSNP and the top ranked nSNP:

D_min_(Z_T_) = min_*rSNP*_(*Z*_*T*_) - max_*nSNP*_(*Z*_*T*_).

Thus, D_min_(Z_T_) is positive when the lowest ranked rSNP is larger than the highest ranked nSNP, and negative when the lowest ranked rSNP is smaller than the highest ranked nSNP.

**Figure 5:**
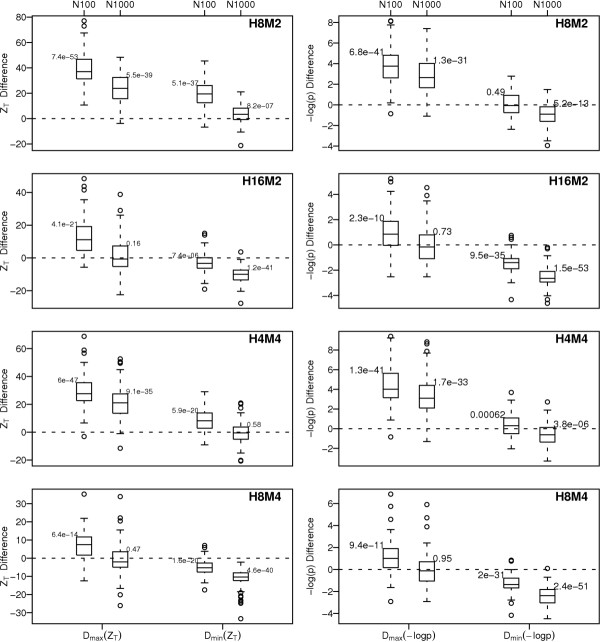
Distribution of difference in importance ZT between the top ranked rSNP and the top ranked nSNP (Dmax(ZT), and lowest ranked rSNP (Dmin(ZT)) and top ranked nSNP. Dmax(-log p) and Dmin(-log p): differences using -log10 p-value from the Fisher exact test. Beside each boxplot is the p-value for the test of whether the mean difference over the 100 replicates is significantly different from 0. Genetic models listed in plot. Analysis design: K4S2, with N100 and N1000 shown on plot.

We computed the analogous quantities, D_max_(-log p) and D_min_(-log p), for the -log_10 _transformed Fisher Exact test p-values. In Figure 5, we have plotted box plots of these differences for several models using analysis designs K4S2N100 and K4S2N1000. P-values for a paired T-test of whether the mean difference is equal to 0 are also placed on the plot. For H8M2, the lowest ranking rSNP has Z_T _that is significantly higher than the highest ranking nSNP for both N100 and N1000, while the difference in -log10(p) is not significantly different from 0 for N100, and is significantly less than 0 for N1000. These plots illustrate that the positive differences are typically more extreme for Z_T _than for -log p, and that the negative differences are less extreme.

## Discussion

A key advantage of the random forest approach is that the investigator does not have to propose a model of any kind. This is important in an initial genome-wide or candidate region association study, where little is known about the genetic architecture of the trait. If interactions among SNPs exist, they will be exploited within the trees, and the variable importance scores will reflect the interactions. Therefore, we expect that when unknown interactions between true risk SNPs exist, the random forest approach to screening large numbers of SNPs will outperform a univariate ranking method in finding the risk SNPs among a large number of irrelevant SNPs. Our genetic models for simulation feature both multiplicative interaction and genetic heterogeneity. The multiplicative interaction results in a marginal effect in the population, the size of which is dependent on many factors, including the amount of heterogeneity. Thus, we have highlighted models in which univariate tests still have power, and shown that the random forest analysis can outperform these tests for selecting subsets of SNPs for further study. For models with genetic heterogeneity and interactions resulting in no main effect, similar to the models described by Ritchie et al. [10], the performance of random forests compares considerably more favorably to univariate tests (data not shown), since the univariate tests have no power when main effects are absent. Further investigation of how to determine a cutoff for SNPs to keep for further analysis is needed. Unfortunately, this task is likely to be strongly dependent on information that is impossible for an investigator to know *a priori*, such as the underlying genetic model and the ratio of associated risk SNPs to noise SNPs in an analysis.

Our results from analyses with four risk SNPs among 1000 SNPs suggest that even when a high proportion of the analyzed SNPs are unassociated, a random forest can rank interacting SNPs considerably higher than a univariate test, and that the proportional difference in importance between the risk SNPs and the best of the noise SNPs can be larger on average for a random forest. In our scale-up from 100 to 1000 total SNPs, we kept the number of risk SNPs constant. In practice, as we increase the number of SNPs genotyped, we expect that we will also increase the number of risk SNPs (or SNPs in linkage disequilibrium with risk SNPs) that are captured in an analysis. Thus, as a larger and large proportion of the genome or candidate region is captured by a scan, the more likely we will be to have all or most of sets of SNPs that interact, and thus the more likely we are to be in situations where random forest screening will outperform univariate screening of SNP data.

It is important to consider the tuning parameters for such analyses. Consistent with the recommendations made by Breiman and Cutler [22,29], the number of variables randomly selected at each split seems to have minimal effect over a wide range of values surrounding the square root of the number of covariates (SNPs). Breiman and Cutler do not recommend a method to determine the number of trees necessary for an analysis. The documentation examples typically use on the order of 100–1000 trees, but these examples are primarily in the context of prediction, without computing estimates of variable importance. In our experience with the simulated data sets presented here, in which the truly associated covariates are outnumbered considerably by those that are noise, multiple thousands of trees must be used in order to get stable estimates of the variable importance. In practice, we recommend building several forests for a data set with a given number of trees. If the ranking of variables by importance does not change significantly from forest to forest, then the number of trees is adequate.

We have examined the use of random forests in the context of association studies for complex disease with uncorrelated SNP predictors. Random forests can also be used when predictors are correlated, as is the case with multiple SNPs in linkage disequilibrium within a small genetic region. For any analysis procedure, the more highly correlated variables are, the more they can serve as surrogates for each other, weakening the evidence for association for any one of the correlated variables to the outcome. In a random forest analysis, limited simulations suggest that correlated variables lead to diminished variable importance for each correlated risk SNP (data not shown). One way to limit the problems presented by SNPs in linkage disequilibrium is to use haplotypes instead of SNPs as predictor variables in a random forest. Future challenges include quantifying more completely the effect of linkage disequilibrium among SNPs submitted to a random forest analysis, and developing random forests in the context of haplotypes.

## Conclusions

With the increasing size of association studies, two-stage analyses, in which in the first stage a subset of the loci are retained for further analyses, are becoming more common. The most frequently voiced concern for these analyses is that variables that interact to increase disease risk but have minimal main effects in the population will be missed. Random forest analyses address this concern by presenting a summary importance of each SNP that takes into account its interactions with other SNPs. Current implementations of random forests can accommodate up to one thousand of SNPs in one analysis with the computation of importance. Further, there is no reason to restrict the input variables to SNPs. Potential environmental covariates can also be easily accommodated, allowing for SNPs with no strong main effect, but environmental interactions, to be distinguished from unassociated SNPs. We have shown that when unknown interactions among SNPs exist in a data set consisting of hundreds to thousands of SNPs, random forest analysis can be significantly more efficient than standard univariate screening methods in ranking the true disease-associated SNPs highly. After identifying the top-ranked SNPs and other variables, and weeding out those unlikely to be associated with the phenotype, more thorough statistical analyses, including model building procedures, can be performed.

## Methods

### Variable importance

Rather than selecting variables for modeling, a random forest uses all available covariates to predict response. Here, we use measures of variable importance to determine which covariates (SNPs, in our case) or sets of covariates are important in the prediction. Breiman [22] proposed to quantify the importance of a predictor variable by disrupting the dependence between the variable and the response and measuring the change in the tree votes compared to the original observations. In practice, this is achieved by permuting the variable values among all observations in the out-of-bag sample of each tree. If the variable is predictive of the response, it will be present in a large proportion of trees and be near the root of those trees. Observations with a changed variable value may be directed to the wrong side of the tree, leading to vote changes from the right to the wrong class. Conversely, if the variable is not related to the response, it will be present in few trees and, when present, it will be near a terminal node, so that few tree votes will be changed. In Random Forests (version 5) Breiman and Cutler [29] define the importance index as follows. For individual i, let **X**_i _represent the vector of predictor variable values, y_i _its true class, V_j_(**X**_i_) the vote of tree j and t_ij _an indicator taking value 1 when individual i is out-of-bag for tree j and 0 otherwise. Let **X**^(A,j) ^= (**X**_1_^(A,j)^,..., **X**_N_^(A,j)^) represent the vector of predictor variables with the value of variable **A **randomly permuted among the out-of-bag observations for tree j, and **X**^(A) ^the collection of **X**^(A,j) ^for all trees where N is the total number of individuals in the sample. Letting 1(C) denote the indicator function taking value 1 when the condition C is true and 0 otherwise, the importance index averages over the trees of the forest, and is defined as:



where *N*_*j *_represents the number of out-of-bag individuals for tree j and T is the total number of trees.

The importance index can be standardized by dividing by a standard error derived from the between-tree variance of the raw index *I*_*T*_, . The standardized index is defined as:



The variance  represents the tree to tree variance of *I*_*T*_, rather than the variance of *I*_*T *_due to the sampling of the individuals from a population: the magnitude of *Z*_*T *_increases with the number of trees in the forest, and the number of trees is limited only by computing time. Thus, this standardized index cannot be treated as a Z-score in the traditional sense.

### Simulation models and methods

For simplicity, assume each locus has the same effect, and let (q_0_, q_1_, q_2_) represent the penetrance factors for 0, 1, or 2 risk alleles for an individual locus in a given model. Let G = {g_11_, g_12_, . ., g_HM_} be the multilocus genotype for an individual, where g_hm_(=0, 1, 2 risk alleles) indicates the individual's genotype at locus m (=1, . ., M) of heterogeneity system h (=1, . ., H). Then the penetrance for genotype G is defined as:



For example, for model H2M2, an individual with genotype G = {0101} would have penetrance *P*_*G *_= 1 - (1 - *q*_0_*q*_1_)^2 ^= 2*q*_0_*q*_1 _- (*q*_0_*q*_1_)^2^.

The penetrance factors (q_0_, q_1_, q_2_) and risk allele frequencies, as well as other features of our genetic models, are listed in Table 1. For a given model type, such as H4M4, and a given *λ*_s _and *K*_*p*_, there is a unique allele frequency when we make the assumption that each SNP subunit has equal effect (the given penetrance factor vector) in the population. We chose penetrance factors such that the risk alleles at each locus for the H2M2, H4M2, H8M2, and H16M2 models are approximately additive in effect on the penetrance factor scale. For H4M4 and H8M4, we chose penetrance factor vectors such that the risk alleles show a moderate degree of dominance.

The marginal genotype relative risks (GRRs) listed in Table 1 are the relative penetrances for heterozygote and homozygote carriers of each risk allele, as compared to non-carriers in the population, which would be observed if only a single rSNP were considered at a time. Thus, this is a measure of the observed effect size of each of the rSNPs in the population. The marginal GRRs are modest, in line with what might be expected when there are a large number of small effects contributing to a complex phenotype. For cases, the genotypes for pairs/quartets of SNPs within an interacting system are positively correlated, while SNPs from distinct systems are negatively correlated. The magnitude of the correlations decreases with increasing number of heterogeneity systems and increasing number of equal-effect SNPs interacting within each system.

### Analysis

All analyses were performed on 100 replicate data sets of 500 cases and 500 controls. We treated the SNPs as ordinal predictors. Random forests have one primary tuning parameter: "mtry" the number of randomly picked covariates to choose among for each split. The Random Forest v5 manual [29] recommends trying the square root of the number of predictors, along with values smaller and larger than the square root, and choosing the value that minimizes the out of bag prediction error rate. We considered both the prediction error and the stability of the variable importance estimates when determining the values of mtry to use and the number of trees to grow. We found that the prediction error rate was very stable over a wide range of mtry for the number of trees we required for consistent measures of importance. We analyzed each replicate data set with 4–16 rSNPs and 96 nSNPs using a random forest of 5000 trees, choosing the best split from among a different randomly-selected set of 35 SNPs at each node (mtry = 35). On average, each replicate data set with 100 total SNPs took 40 minutes to complete on a 2.6 Ghz Intel Xeon processor. For data sets with 4 rSNPs and 996 nSNPs (1000 SNPs total), we used 15000 trees, and chose from among 125 SNPs at each node (mtry = 125). Analysis of each replicate of these data sets took 123 minutes on average. User time could potentially be substantially decreased by parallel-processing: trees could be grown on separate nodes, and combined for analysis of importance. However, parallel tree-building is not yet available in the Random Forest progream. To compare the performance of random forests with that of a univariate, one-SNP-at-a-time approach, we tested for association between genotypes for each individual SNP and disease status using a Fisher Exact test [32].

### Ranking of rSNPs

The random forest analysis produces the raw and standardized importance indices (*I*_*T*_, *Z*_*T*_,), which can be used to rank order the importance of SNPs much as p-values from association tests are used. Using either method, the ranking of the SNPs in an analysis can be used to prioritize which sets of SNPs (or genome regions) should be followed up with further genotyping and/or additional analyses. We use the convention that a rank of 1 is the highest ranking SNP.

We compare the ranking of the raw and standardized importance measures, and further compare these with the rank based on p-values from a test of association, the Fisher Exact test, to determine whether the random forest can better discriminate susceptibility SNPs from SNPs unrelated to disease status when there is interaction and heterogeneity among and between SNPs.

## Authors' contributions

KLL conceived of and led the design of the study, coordinated all phases of simulation and analysis, and drafted the manuscript. LBH and JS automated the simulation and random forest analysis procedures, and participated in the design and analysis of study results. PVE added critical insight to the development of the genetic models and participated in the interpretation of study results. All authors provided comments on a draft manuscript and read and approved the final manuscript.
